# Anatomical Variations of the Circulus Arteriosus in Cadaveric Human Brains

**DOI:** 10.1155/2014/687281

**Published:** 2014-05-07

**Authors:** S. A. Gunnal, M. S. Farooqui, R. N. Wabale

**Affiliations:** Department of Anatomy, Rural Medical College, Post Loni Tal Rahata District Ahmednagar, Maharashtra 413736, India

## Abstract

*Objective*. Circulus arteriosus/circle of Willis (CW) is a polygonal anastomotic channel at the base of the brain which unites the internal carotid and vertebrobasilar system. It maintains the steady and constant supply to the brain. The variations of CW are seen often. The Aim of the present work is to find out the percentage of normal pattern of CW, and the frequency of variations of the CW and to study the morphological and morphometric aspects of all components of CW. *Methods*. Circulus arteriosus of 150 formalin preserved brains were dissected. Dimensions of all the components forming circles were measured. Variations of all the segments were noted and well photographed. The variations such as aplasia, hypoplasia, duplication, fenestrations, and difference in dimensions with opposite segments were noted. The data collected in the study was analyzed. *Results*. Twenty-one different types of CW were found in the present study. Normal and complete CW was found in 60%. CW with gross morphological variations was seen in 40%. Maximum variations were seen in the PCoA followed by the ACoA in 50% and 40%, respectively. *Conclusion*. As it confirms high percentage of variations, all surgical interventions should be preceded by angiography. Awareness of these anatomical variations is important in neurovascular procedures.

## 1. Introduction

Brain constitutes only 2% of the body weight but it requires 25% of oxygen, contents from each single breath. Brain is highly susceptible to lack of oxygen and blood supply; the blood supply to the brain is provided by pairs of two vessels, the internal carotid arteries and vertebral arteries. Both the arteries reach at the base of brain and form a circular arterial channel called the circulus arteriosus. Circulus arteriosus was first investigated by the scientist Thomos Willis in 1962. Henceforth, it is also called circle of Willis (CW). Though the name is circular the channel is not exactly circular. It is polygonal in shape. Circle of Willis (CW) is a polygonal anastomotic channel at the base of the brain which unites the internal carotid and vertebrobasilar system of arteries in the interpeduncular fossa. It maintains the steady and constant blood supply to the brain. The variations of circulus arteriosus are seen often. The aim of the present work is to find out the percentage of normal pattern of the CW and the frequency of variations of the CW and to study the morphological and morphometric aspects of all the components of CW separately.

## 2. Materials and Methods

Study was done in the Department of Anatomy Rural Medical College, PIMS, Loni. The study was started by undertaking the institutional ethical clearance (PIMS/PhD/RC/2013/28). Circulus arteriosus was studied on 150 formalin preserved brains of human cadavers. The cadaveric bodies from which brains removed were of unknown age and unknown cause of death. The approximate age was 40–60 years. The brains with the gross morphological variations were excluded from the study. Arachnoid mater in the interpeduncular fossa was removed carefully to expose the circulus arteriosus. All components of the CW were observed. Relations of the CW were noted. Gross morphological variations of CW were noted and photographed. The arterial skeletons of the CW were carefully and delicately separated from brain tissue and pasted on the black plastic sheets for better view. Detailed study of these regarding morphological and morphometric aspects of the circulus arteriosus was done. Length and diameter of all the components forming CW were measured. Morphological variations were well photographed. Specimens were sorted out according to classification of the morphological variation of different components of circulus arteriosus (CW). Variations of all the segments were noted and were photographed. The variations such as hypoplasia, aplasia, duplication, fenestrations, and difference in dimensions with opposite segments were noted. The data collected in the study was analyzed to calculate the mean, standard deviation, and percentage.

## 3. Observations and Results

### 3.1. Different Types of Circulus Arteriosus Were Found in the Present Study (Figure 1)

Out of those, there was one classical type of CW (a) present. Six types were associated with the variations of ACoA ((b1) to (b6)). Three types were related to the variations of Precommunicating segment (A1) of anterior cerebral artery ((c1) to (c3)). Four types were associated with the variations of posterior communicating artery (PCoA) ((d1) to (d4)), five were associated with the variations of precommunicating segment (P1) of posterior cerebral arteries ((e1) to (e5)), and two other types of CW ((f1) and (f2)) were seen. These two types were not associated with any particular boundary of CW.

### 3.2. Normal and Complete Circulus Arteriosus without any Gross Variation Was Found in 90 Cases (60%) (Figures 2(a) and 2(b))

Anteriorly, it is bounded by anterior communicating artery (ACoA). ACoA unites two anterior cerebral arteries (ACA) of either side. ACA of either side forms the anterolateral boundaries of CW. Posteriorly, there is the presence of the bifurcation of the basilar arteries (BA) into two posterior cerebral arteries (PCA) of either side. On either side of CW its lateral boundary is formed by posterior communicating artery (PCoA). Junction of PCoA to the PCA divides the PCA into the proximal P1 precommunicating segment and distal P2 postcommunicating segment, just like the same ACoA divides the ACA into proximal A1 precommunicating segment and distal A2 postcommunicating segment. Lateral corners of CW are formed by the termination of ICA, while the posterior corner is formed by the termination of BA.

Segments of CW are the ACoA, A1 of (b) and (a), PCoA of (b) and (a), and P1 of (b) and (a).

### 3.3. Morphological Variations of ACoA Were Seen in 60 (40%)

**Figure 1:**
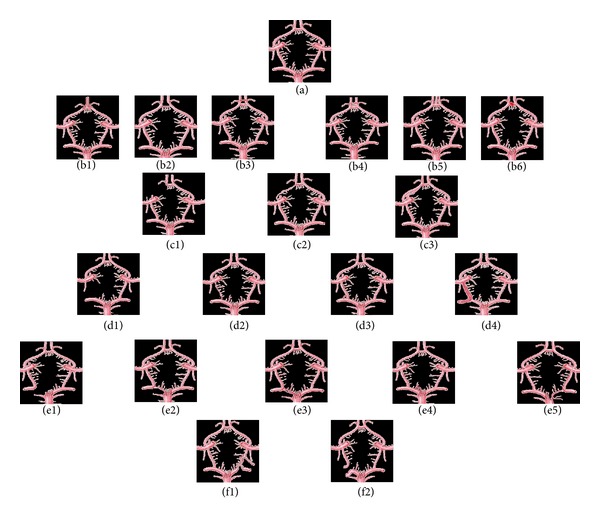
Twenty one different types of circulus arteriosus found in the present study. (a) Classical type of CW (1). (b) Six types associated with the variations of ACoA (6). (c) Three types associated with the variations of A1 (3). (d) Four types associated with the variations of PCoA (4). (e) Types associated with the variations of P1 (5). (f) Other types of CW were seen in (2).

Variations found were the absence of ACoA. Thin hypoplastic ACoA, double ACoA, fenestrated ACoA, third A2 arising from ACoA and ACoA associated with aneurysm were found (Figure 1(b1)–(b6)).

**Figure 3:**
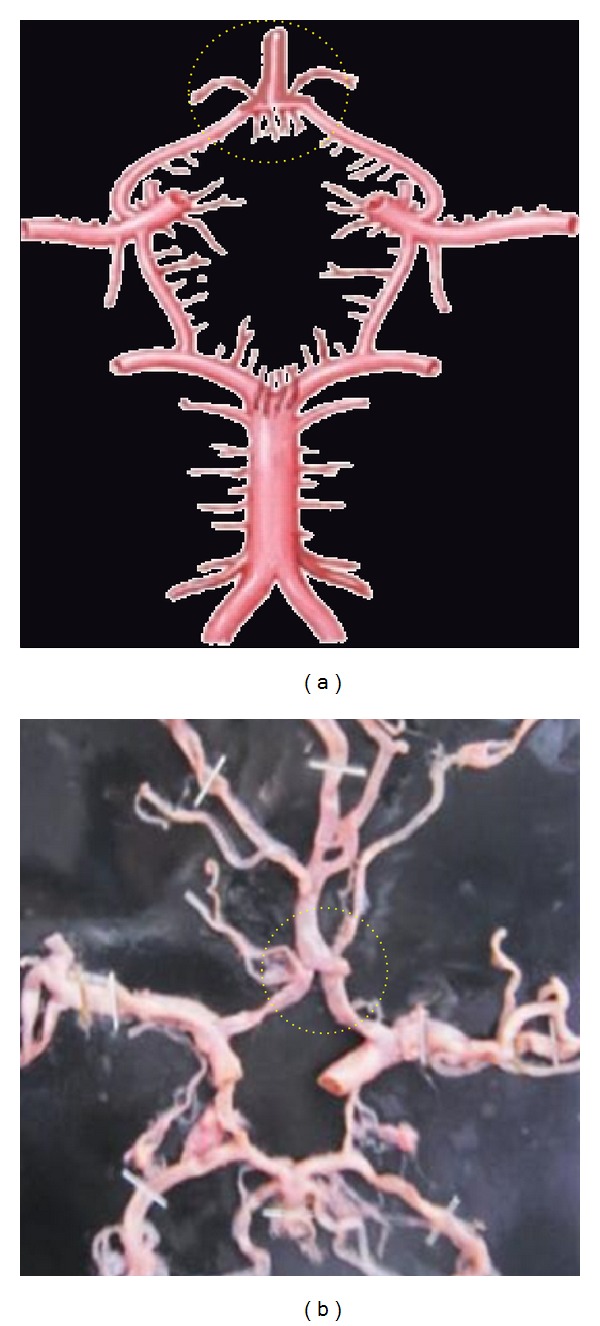
Absence of ACoA. Dotted circle is showing the aplasia of ACoA replaced by the azygos ACA.

**Figure 4:**
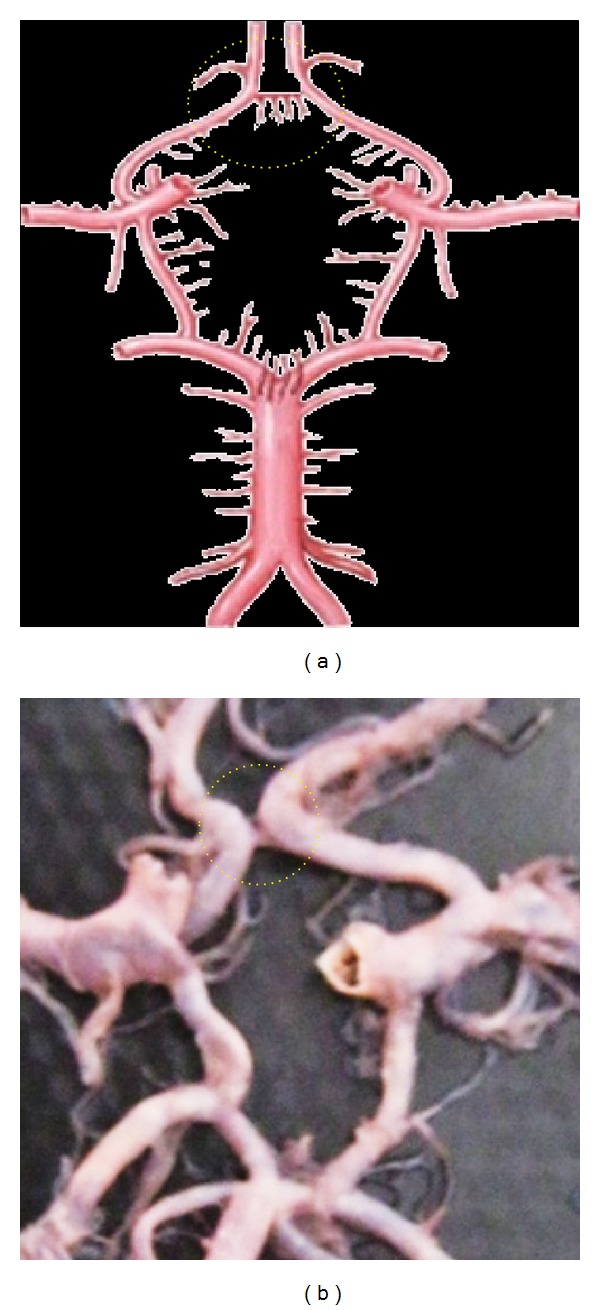
Hypoplastic ACoA shown by dotted circle.

**Figure 5:**
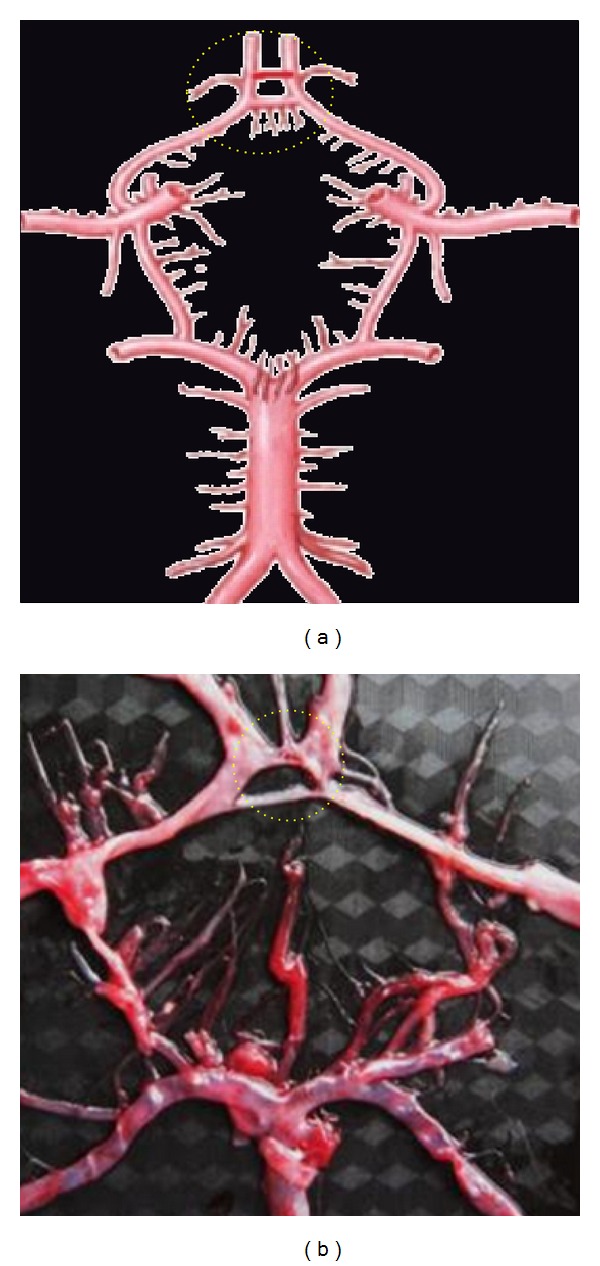
Double of ACoA shown by dotted circle.

**Figure 6:**
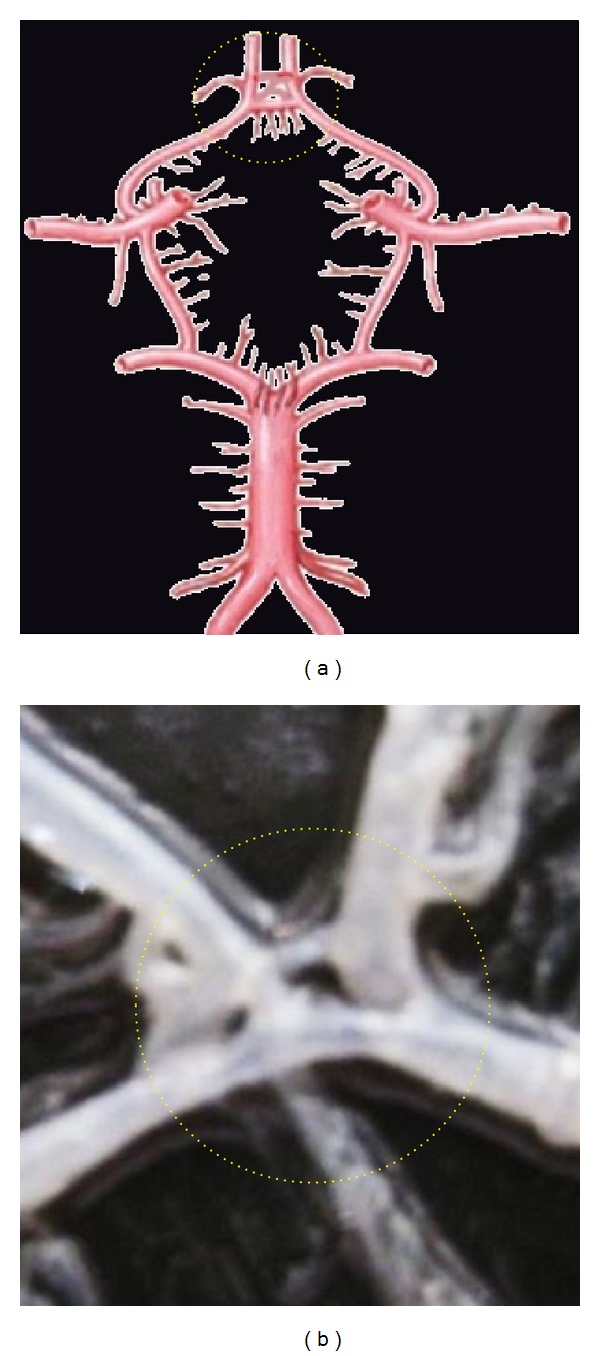
Fenestrated ACoA shown by dotted circle.

**Figure 7:**
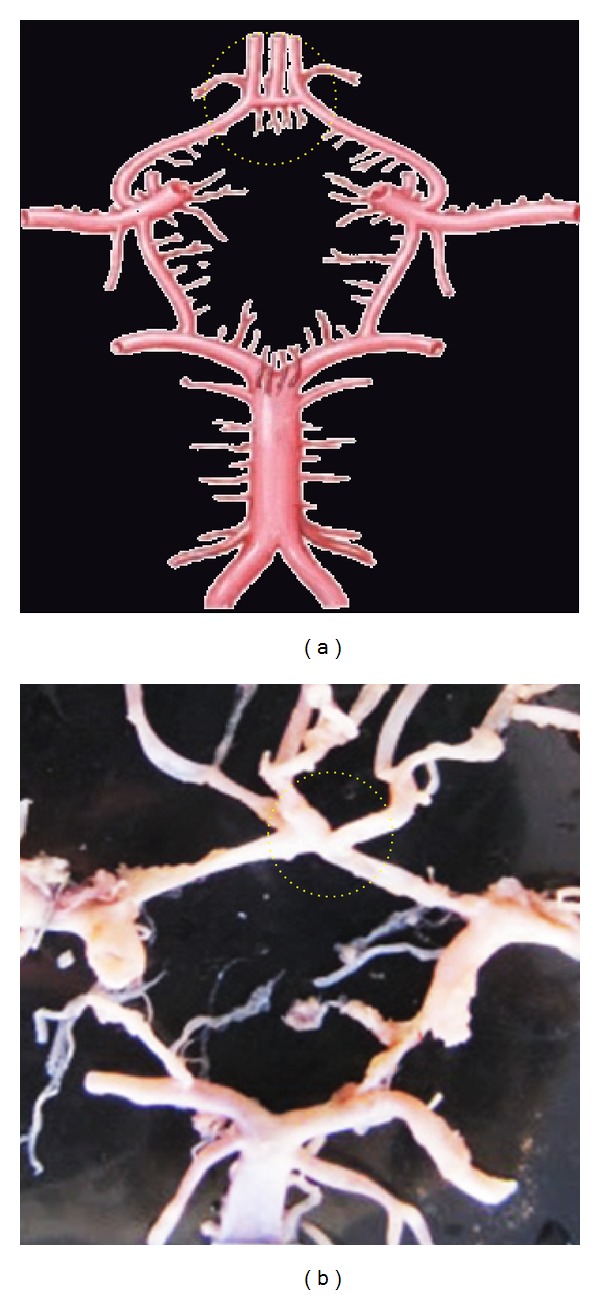
Third median A2 from ACoA shown by dotted circle.

**Figure 8:**
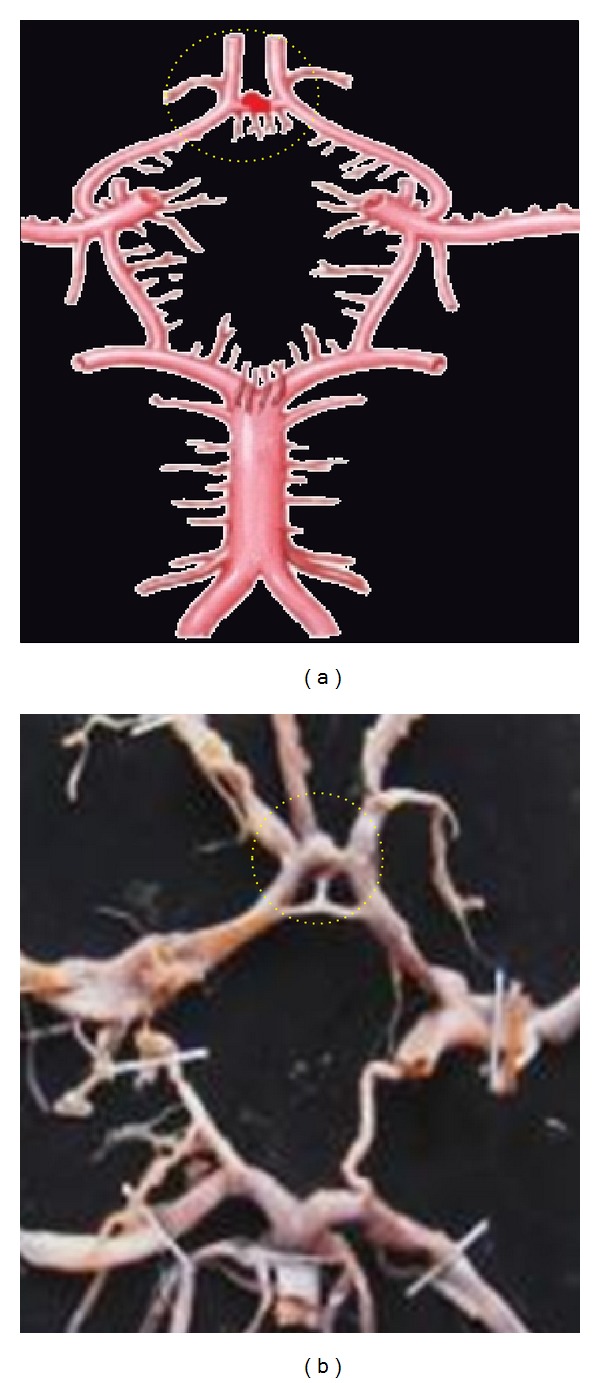
Aneurysm associated with ACoA shown by dotted circle.

Absence of ACoA was seen in 12 (8%) cases. In those cases, anterior boundary of CW was formed by azygos ACA instead of ACoA (Figure 3). Thin hypoplastic ACoA was seen in 10 cases (6.66%) (Figure 4). Double ACoA was seen in 16 (10.66%) cases. In those cases anterior boundary of CW was formed by two ACoA (Figure 5). Fenestrated ACoA was seen in 5 cases (3.33%) (Figure 6). Third median A2 was arising from ACoA and was seen in 5 cases (3.33%) (Figure 7). Aneurysm associated with ACoA was seen in 12 (8%) cases (Figure 8).

### 3.4. Morphological Variations of A1 Were Present in 21 (14%)

Variations noted were Aplasia, Hypoplasia and Duplication (Figure 1(c1)–(c3)).

**Figure 9:**
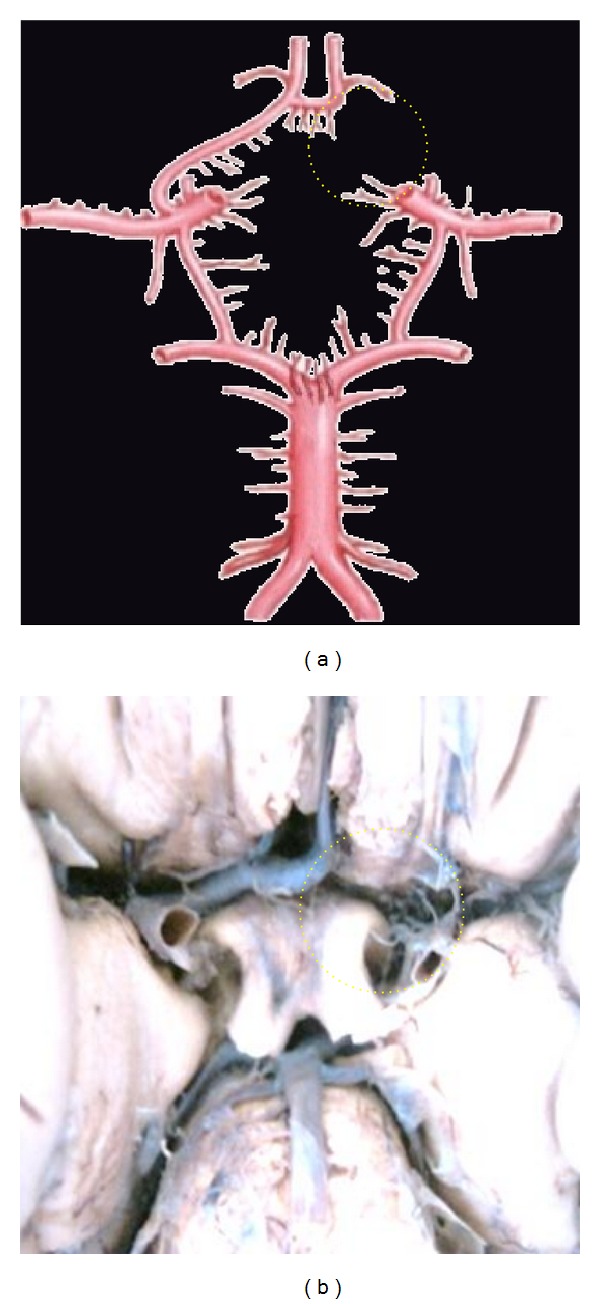
Aplasia of A1 shown by dotted circle.

**Figure 10:**
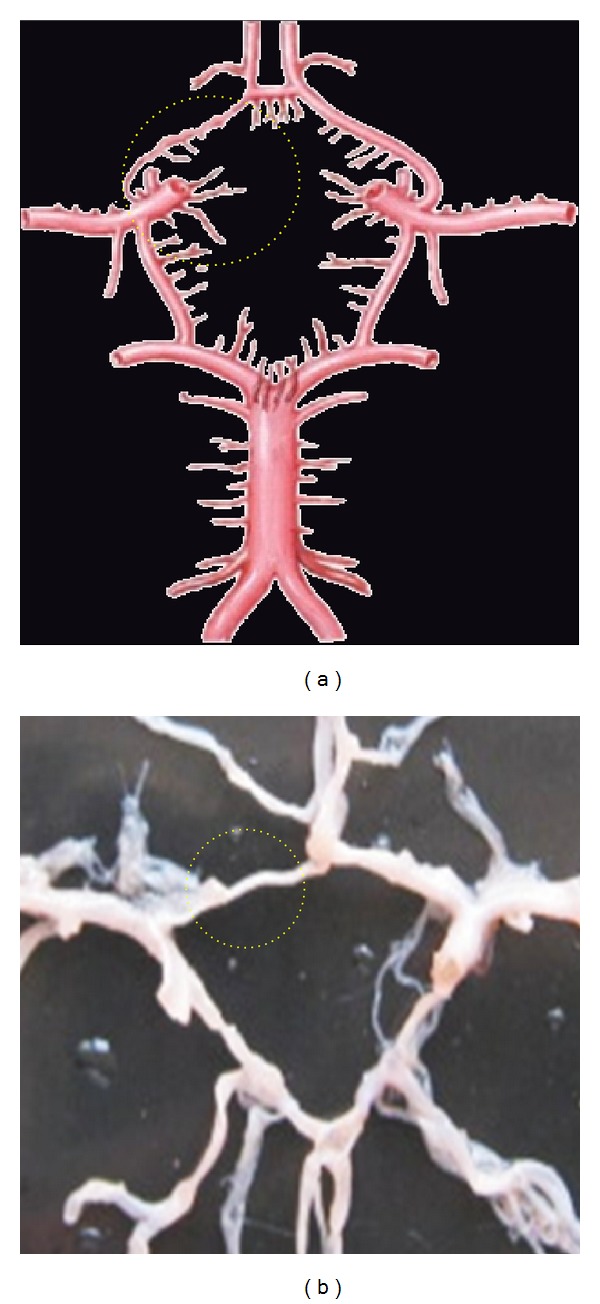
Hypoplasia of A1 shown by dotted circle.

**Figure 11:**
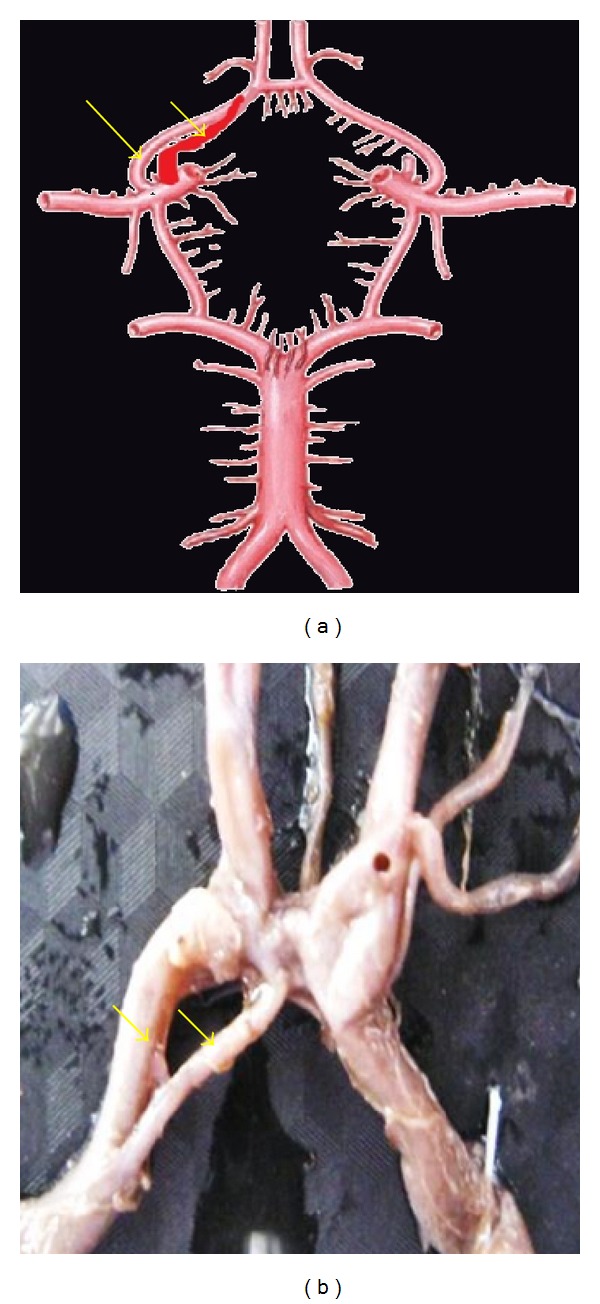
Duplication of A1 shown by two arrows.

Aplasia was seen in 4 cases (2.66%) (Figure 9). In these cases, one of the boundaries of the circle was absent. Hypoplasia was seen in 8 cases (5.33%) (Figure 10). Even though all boundaries of CW were present, there are chances of defective circulation due to hypoplasia of arteries. Duplicated A1 was seen in 9 cases (6%) (Figure 11).

### 3.5. Morphological Variations of PCoA Were Seen in 75 (50%)

Main variations found were aplasia, hypoplasia, fenestration, and foetal pattern of P2 segment which was noted (Figure 1(d1)–(d4)).

**Figure 12:**
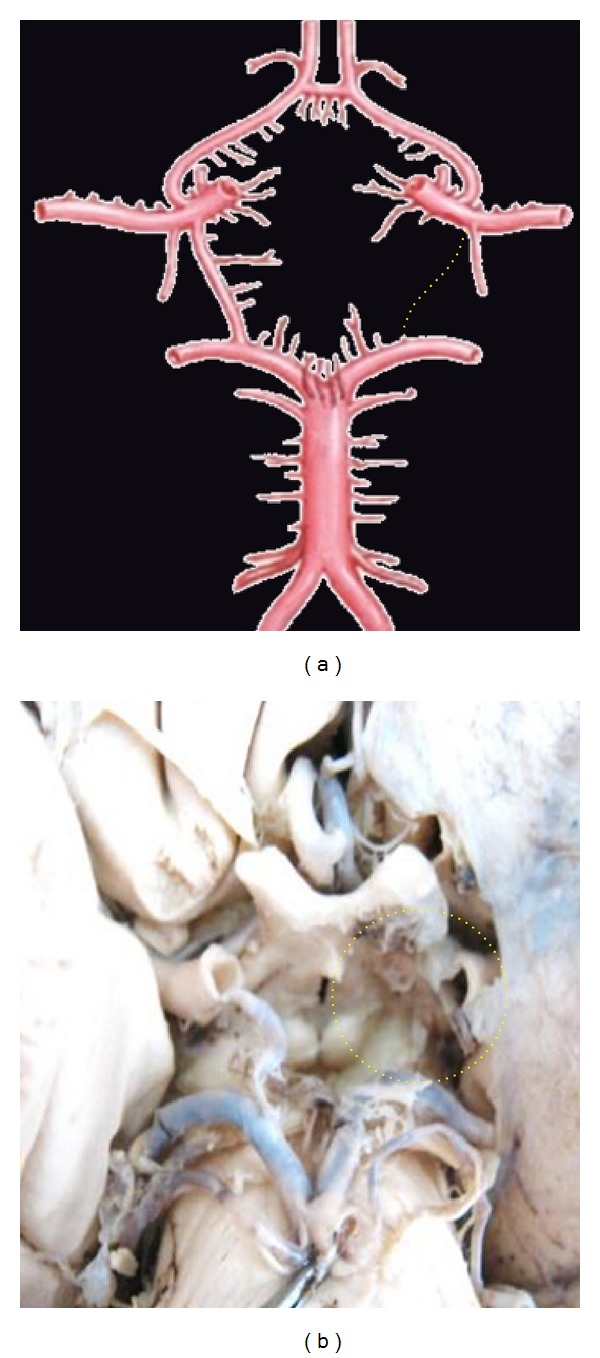
Aplasia of PCoA shown by dotted circle.

**Figure 13:**
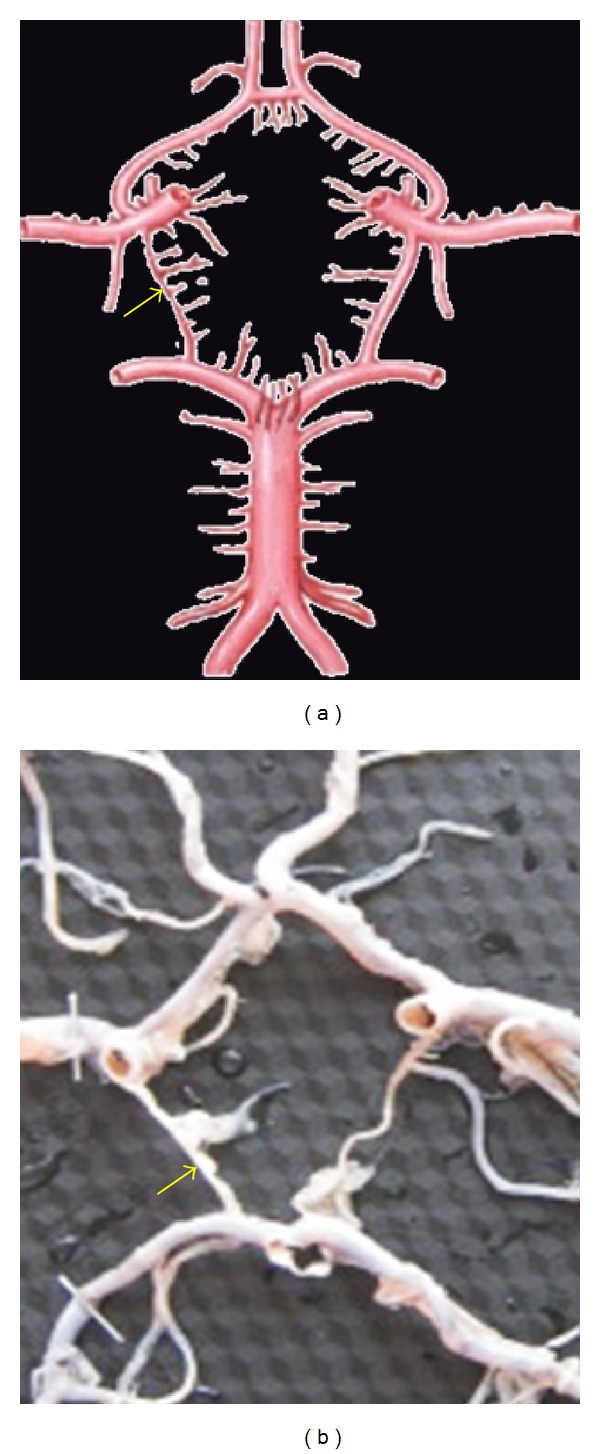
Hypoplasia of PCoA shown by arrow.

Aplasia was seen in 6 cases (4%) (Figure 12) Hypoplasia was seen in 41cases (27.33%) (Figure 13).

**Figure 14:**
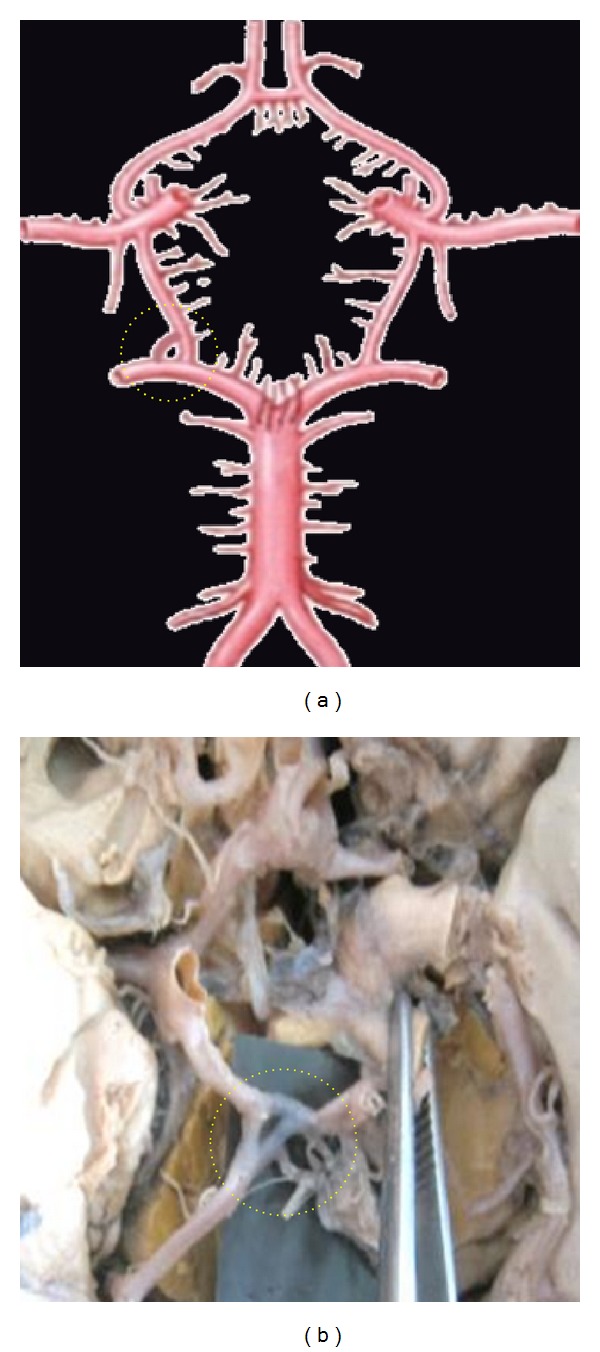
Fenestration of PCoA shown by dotted circle.

**Figure 15:**
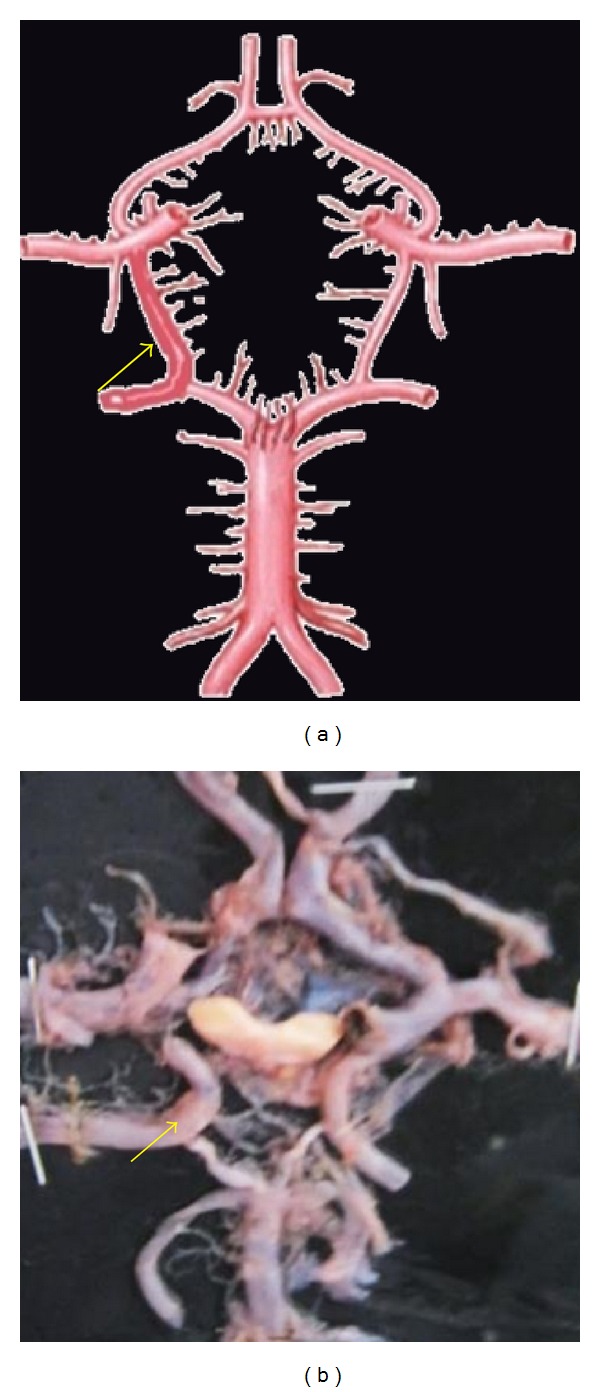
Remnant of fetal pattern of PCoA shown by arrow.

Fenestration was seen in 1 case (0.66%) (Figure 14) (remnant of foetal pattern giving main contribution in the formation of P2 was seen in 27 cases (18%) (Figure 15)).

### 3.6. Morphological Variations of P1 Were Seen in 21 (14%)

Variations found were aplasia, hypoplasia, fenestration, and duplication and the common stem of origin with SCA was seen (Figure 1(e1)–(e5)).

**Figure 16:**
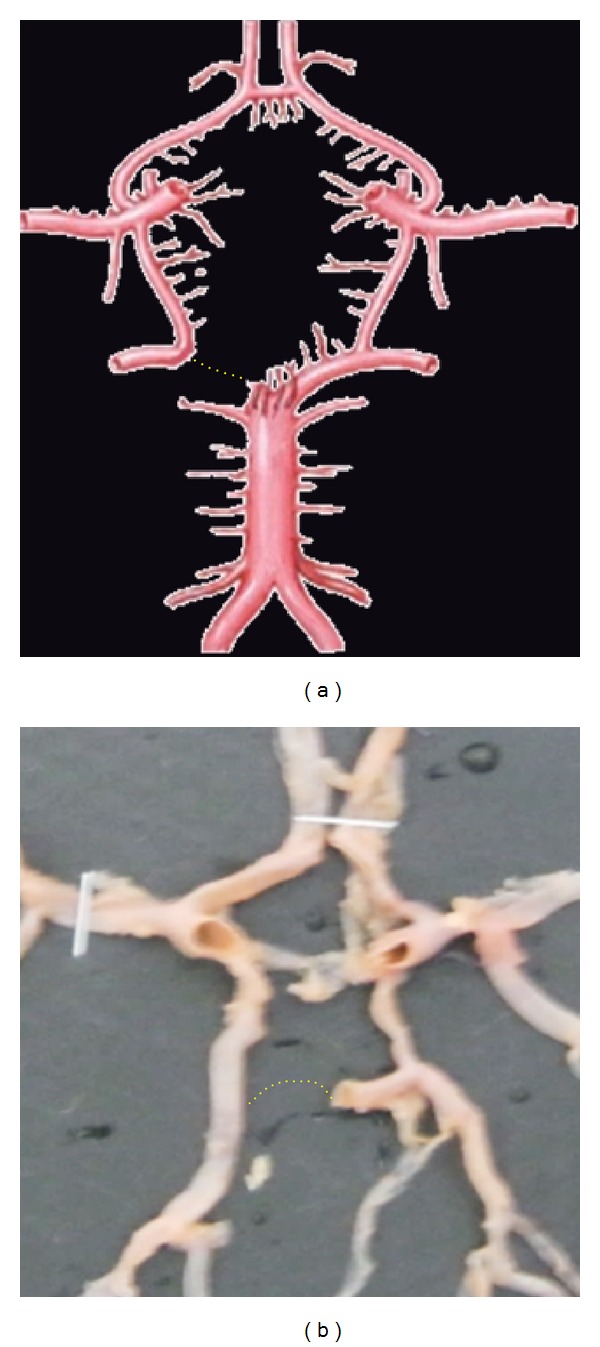
Aplasia of P1 shown by dotted line.

**Figure 17:**
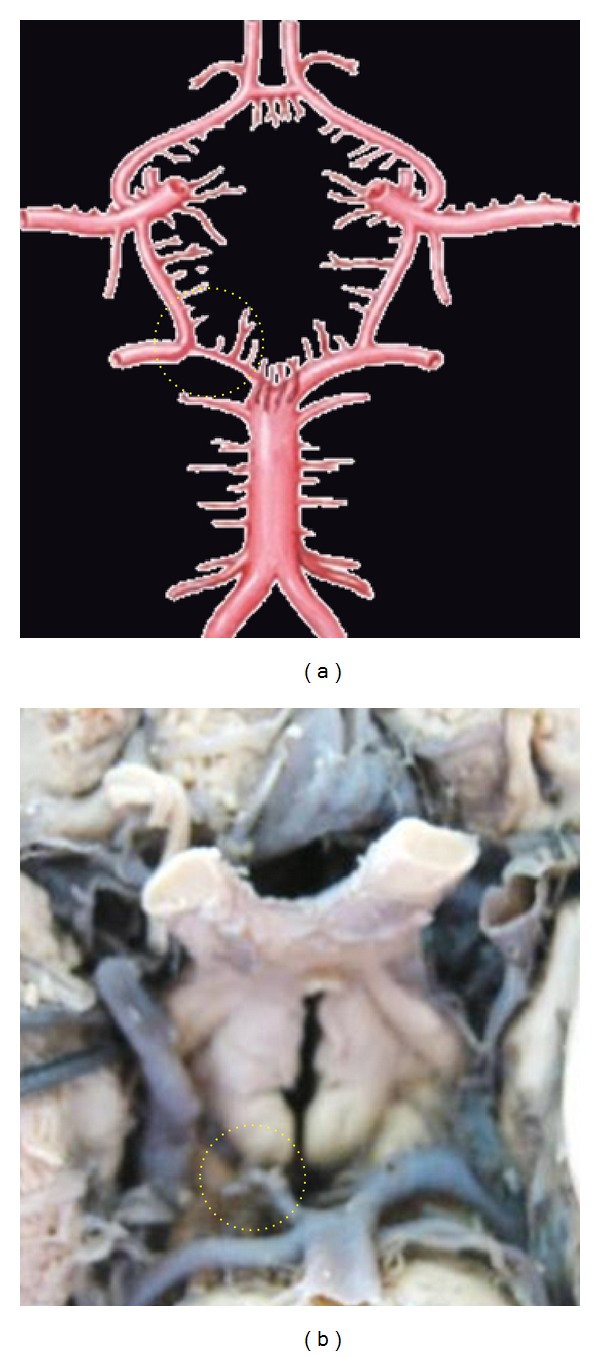
Hypoplasia of P1 shown by dotted circle.

Aplasia was seen in 4 cases (2.66%) (Figure 16) Hypoplasia was seen in 9 cases (6%) (Figure 17).

**Figure 18:**
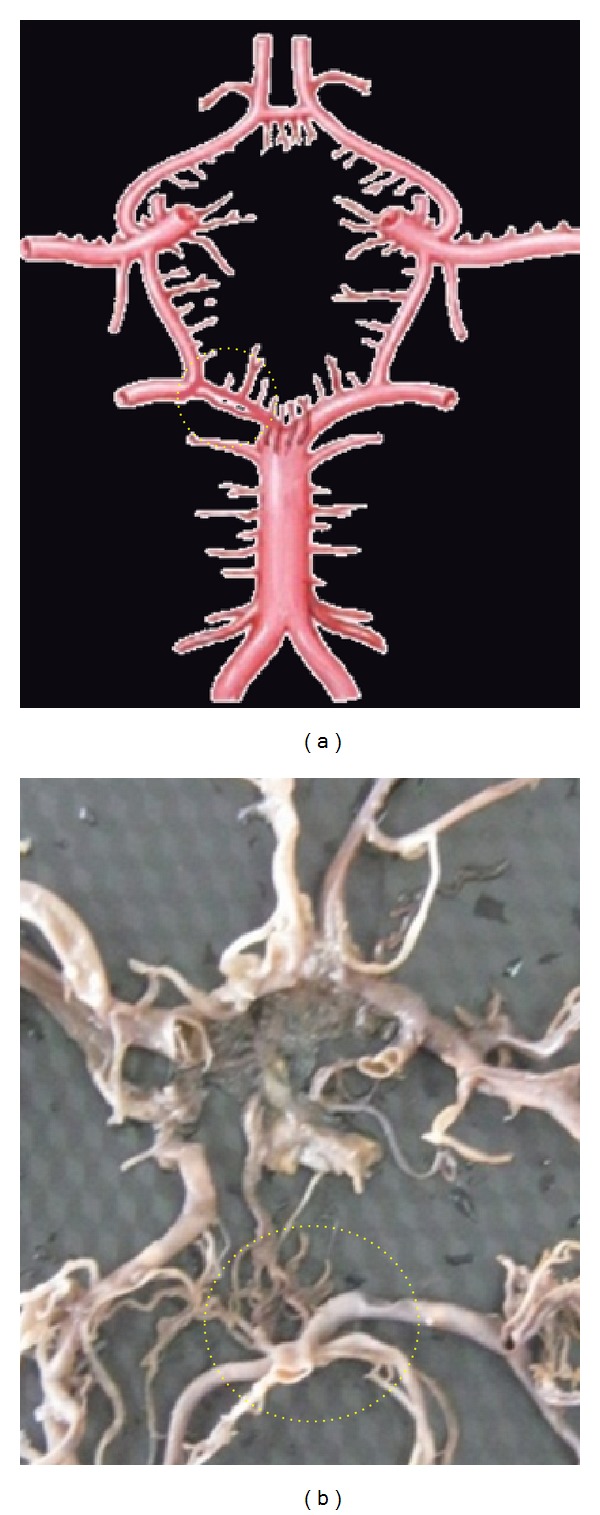
Duplication of P1 shown by dotted circle.

**Figure 19:**
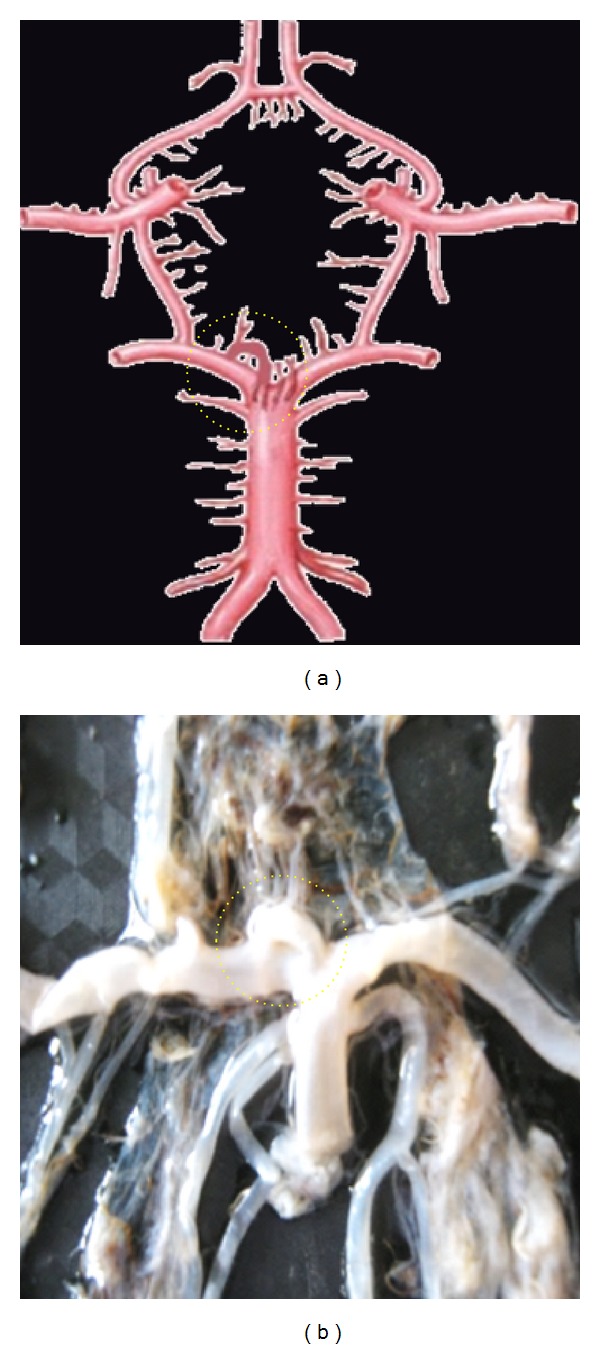
Fenestration of P1 shown by dotted circle.

Duplication was seen in 3 cases(2%) (Figure 18). Fenestration was seen in 2 cases (1.33%) (Figure 19).

**Figure 20:**
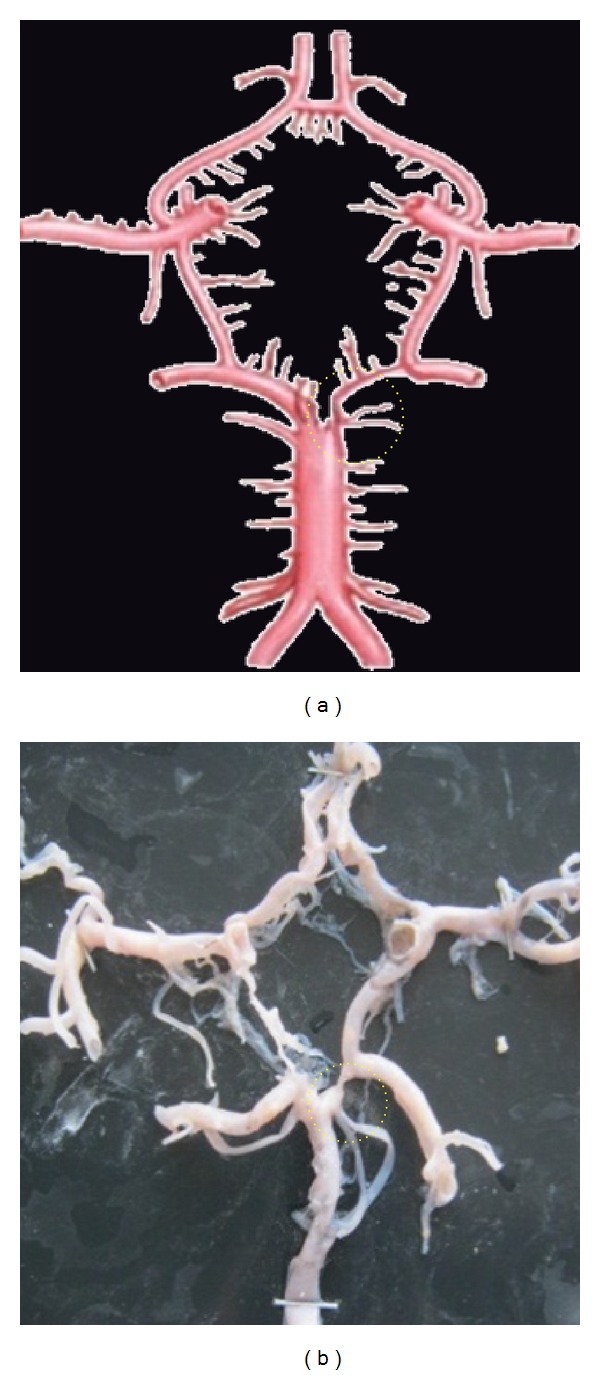
Common stem of origin with SCA and P1 shown by dotted circle.

Common stem of origin with SCA wase seen in 3 cases (2%) (Figure 20).

### 3.7. Other Types of Variations of CW Were Seen in Three Cases (2%) (Figure 1(f1)-(f2))

**Figure 21:**
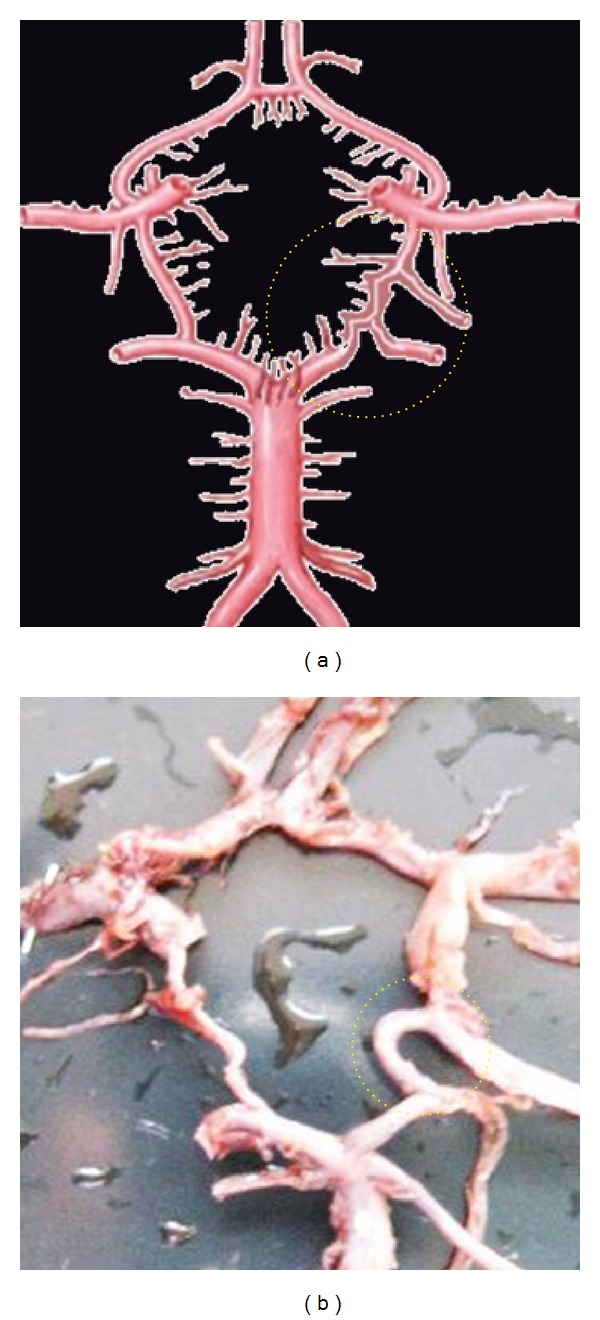
First type in other variation of cw shown by dotted circle.

First type (Figure 21): in the first type, there was presence of an extrasegment in the boundary of CW. The extrasegment was present in between the PCA and PCoA. From either end of the extrasegment, there was the origin of the P2 segment of the PCA. This type was found in only one specimen in the present study (0.66%).

**Figure 22:**
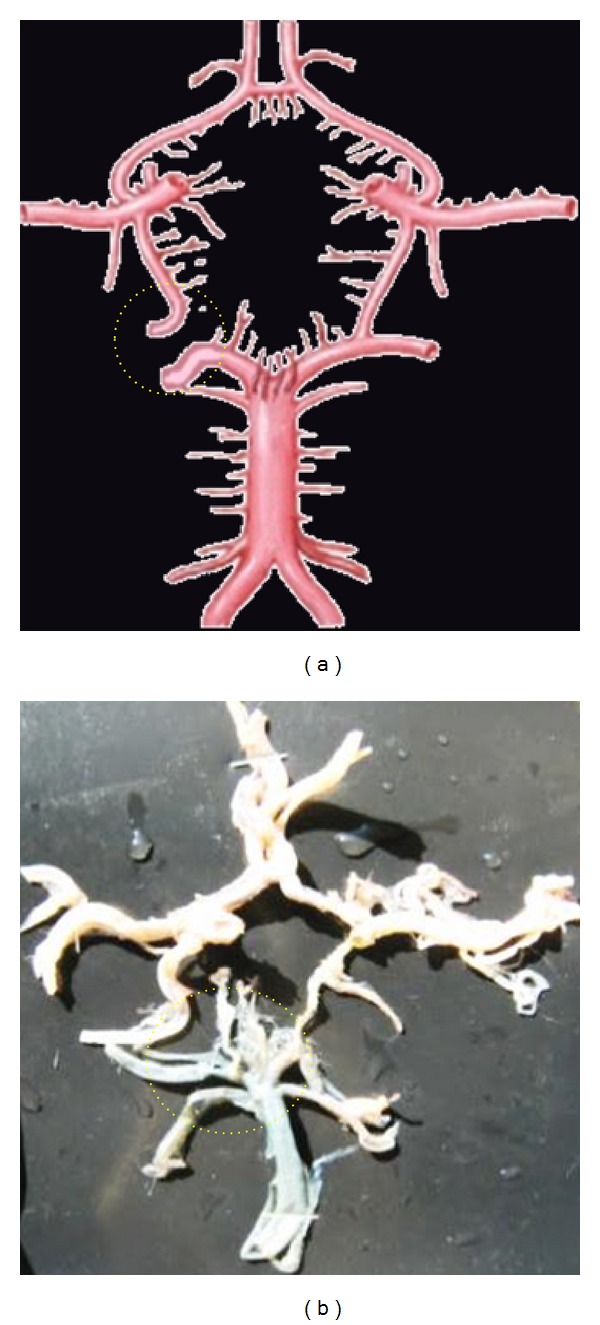
Second types in other variation of cw shown by dotted circle.

Second type (Figure 22): in the second type of variations, even though all the components required for the formation of CW were present, circle was incomplete as the PCoA was not uniting with the PCA to form P1. Therefore, these two segments were separate without communication with each other and because these two separate P2 segments of PCA were formed. This type was seen in 2 cases (1.33%).

Morphological variations seen were tabulated as per the different segments (Table 1).

Along with the morphological variations, morphometric observations of all the components of CW were done and tabulated (Table 2).

**Figure 23:**
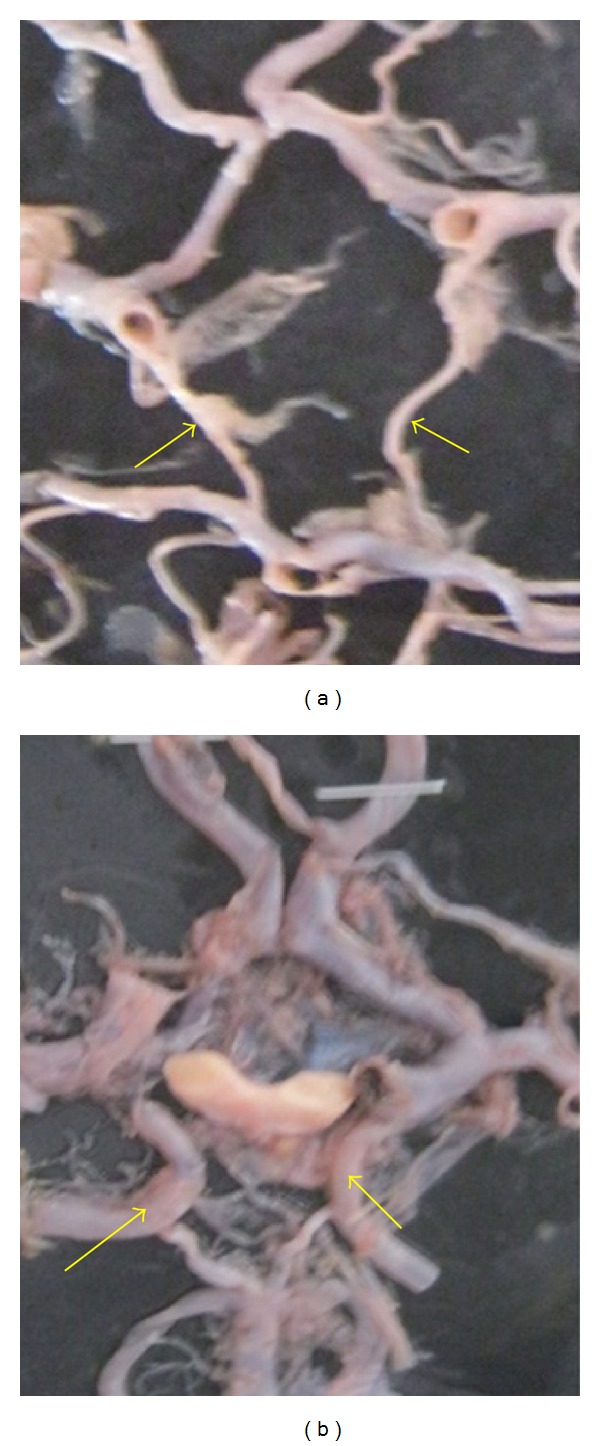
Adult type and fetal type of PCoA. (a) Adult type and (b) fetal type shown by arrow.

In addition to the morphometric observations according to gross comparative size PCoA and P2 segment of PCA, CW was divided into the adult type and fetal type (Figures 23(a) and 23(b)).

If the P2 segment of PCA is equal in diameter of the PCoA or P2 is continuation of PCoA, it was categorized as an foetal type.

If P1 and P2 segments are more or less equal in diameter and PCoA is smaller in diameter than P1 and P2, it was considered as an adult type.

In the present study, adult type of PCoA artery was seen in 116 cases (77.33%). Foetal type of PCoA was seen in 27 cases (18%). In the rest of 7 cases, it was absent.

**Figure 24:**
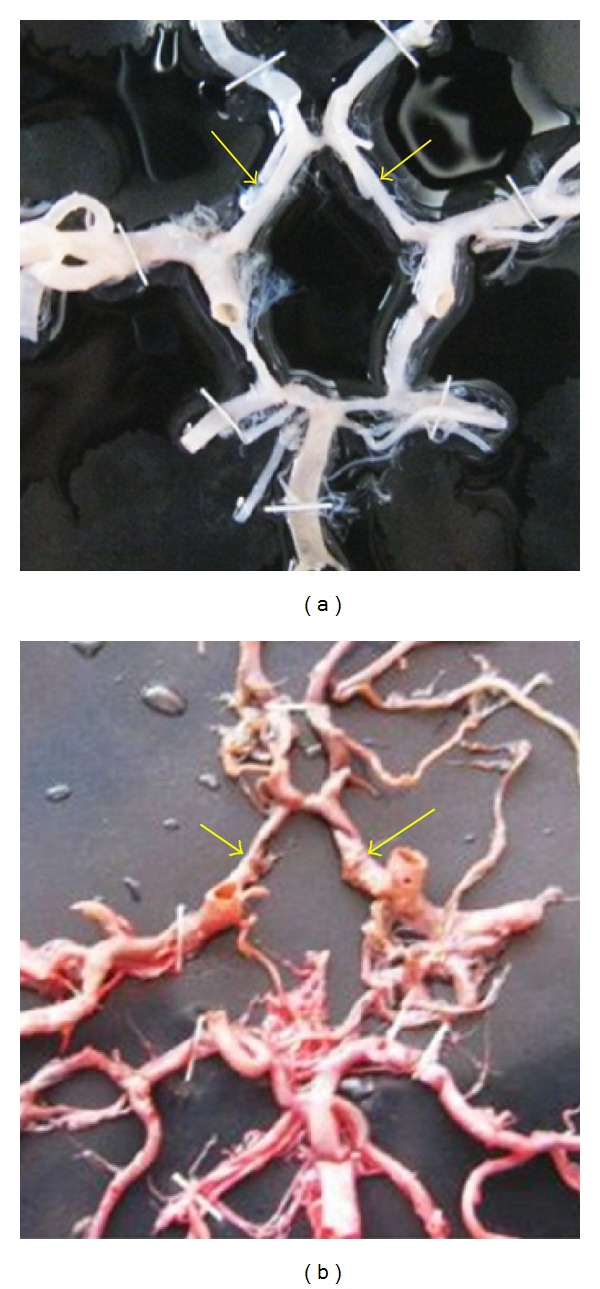
Symmetry of A1 shown by arrows. (a) Symmetrical and (b) asymmetrical.

**Figure 25:**
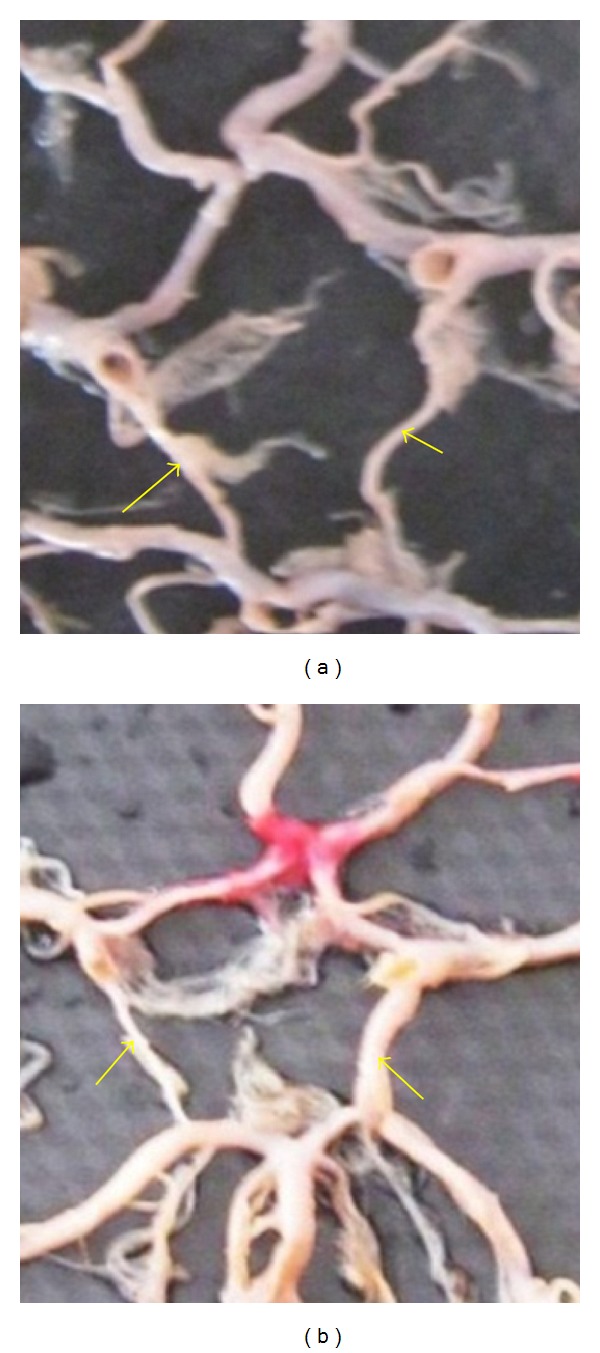
Symmetry of PCoA shown by arrows. (a) Symmetrical and (b) asymmetrical.

**Figure 26:**
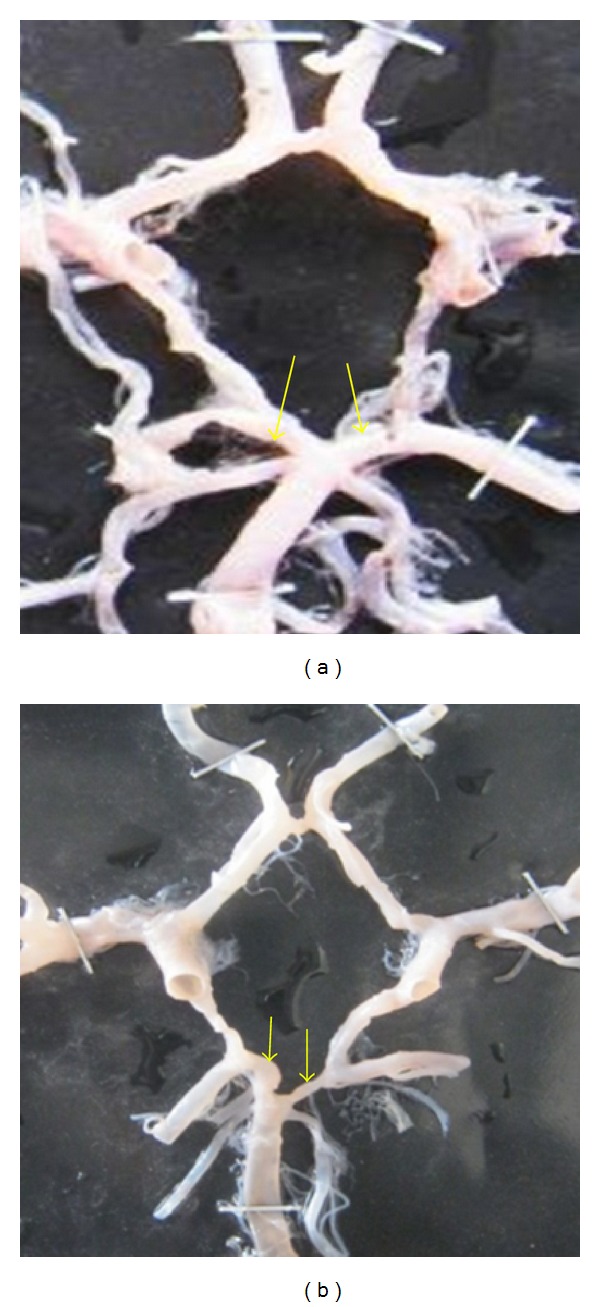
Symmetry of P1 shown by arrows. (a) Symmetrical and (b) asymmetrical.

Symmetry of different components of CW was observed. Symmetric and asymmetric A1 segment is shown in Figure 24; Symmetric and asymmetric PCoA is shown in Figure 25; and Symmetric and asymmetric P1 segment is shown in Figure 26. Percentages of the symmetry were tabulated (Table 3).

Percentages of different variations of aplasia, hypoplasia, duplication, and fenestrations regarding different components were calculated and tabulated (Table 4).

## 4. Discussion

Blood supply to the brain is mainly from the circulus arteriosus and Thomas Willis was pioneer in describing CW in 1962 [1]. Since then, many authors have reported number of anatomical variations in the formation of CW [2, 3].

All these variations are either due to the disappearance of the vessels that normally persist or the persistence of the vessels that normally should disappear or formation of new vessels due to hemodynamic factors. In most of the arterial variations, the brain function may not be affected due to the collateral circulation and compensation from the arteries of the other side [4, 5].

The circle of Willis from 150 adult brains was studied for morphological variations. Twenty one (21) types of CW were found. Findings of present data are compared with the previous studies.  Table 5 shows the comparison of data associated with the morphological variations of CW. The incidence of an incomplete CW in the present study was 10.66%. This incidence extremely varies in the literature. Several authors do not find any incomplete CW [6–8]. Incomplete CW was mostly because of absence of PCoA.

The circle of Willis is not an equalizer and distributor of blood from different sources. There is no mingling of blood from different sources under normal circumstances. It forms an anastomosis and offers a potential shunt under abnormal conditions such as occlusion or spasm [9].

There is a number of morphological studies on each arterial segment of CW. Comparison of morphological variations of ACoA and A1 is done in Tables 6 and 7, respectively.

The common occurrence of anomalies in the anterior communicating vessel is easily appreciated from the studies on the embryology of the CW [7, 10].

Anomalies discovered in CW can be correlated by embryological data. Blood vessels of brain develop from a primitive plexus of vascular channels. Certain vascular channels develop and persist and certain channels disappear in accordance with contemporary needs and relations of the areas to be supplied [11].

All the cerebral arteries are primarily derived by the carotid system. The P2 segment of PCA originates from ICA in initial period of development approximately up to the 14 mm stage of human embryo, subsequently the vertebral-basilar system develops and contributes to the formation of P2. Thread-like PCoA is due to the basis of “atrophy” of the vestigial connecting branch during the developmental process [11]. Hypoplasia of the PCoA is the most commonly found anomaly in the literature. In present study, the incidence of the hypoplastic PCoA was 27.33%. Most of the anatomical variations have been reported on the PCA and PCoA. These are compared with the previous studies in the Tables 8, 9, and 10.

In adults, CW is divided into three different types according to the contribution in formation of P2 segment of PCA joining the P1 segment and PCoA. Three types are the adult configuration, transitional configuration, and fetal or embryonic configuration. In adult configuration, P1 diameter is larger than the PCoA diameter. In transitional configuration, diameter of both is same and their contribution is approximately equal in formation of p2 of PCA. Fetal or embryonic configuration shows smaller diameter of the P1 than diameter of PCoA and P2. In present study we have divided the CW into only adult and the fetal type of configuration (Table 9).

Morphometric studies on CW are comparatively less than the morphological studies. Surgeon's assessment of the feasibility of shunt operations requires the knowledge of the normal size of these vessels. Vessels with 1 mm diameter or less than it were regarded as abnormal [12, 13].

Dimensions of the vessels vary greatly. Hypoplasia was the commonest anomaly in CW. It was mostly seen in PCoA followed by P1 and P2 segments of PCA [12–16]. A significant inverse relationship existed between the diameters of the posterior cerebral and posterior communicating arteries of the same side ensuring better blood supply to that hemisphere. Morphometric observations of CW are compared with the previous studies in Table 11.

The functional significance of the anomalies of the circle of Willis cannot be stated with certainty. In occlusive vascular disease of the brain collateral circulation becomes important. Compensatory circulation will be more effective in the presence of an intact CW than in one in which deficiency is present.

For effective establishment of collateral circulation through CW, PCoA and ACoA play an important role. If one of these arteries is thin thread like, then collateral circulation through the circle may be impaired, partially or completely. For establishment of better collateral circulation, connections between the two sides of the circle of Willis as well as the connections between the internal carotid and vertebrobasilar systems are equally important.

## 5. Conclusion

In the present study complete CW was seen in 60%. Gross variations were present in 40%. Maximum variations were present in the PCoA 50% followed by the ACoA in 33.33%, respectively. As it confirms high percentage of variations in the formation of CW, all surgical interventions should be preceded by angiography. Awareness of these anatomical variations is important in the neurovascular procedures.

## Figures and Tables

**Figure 2:**
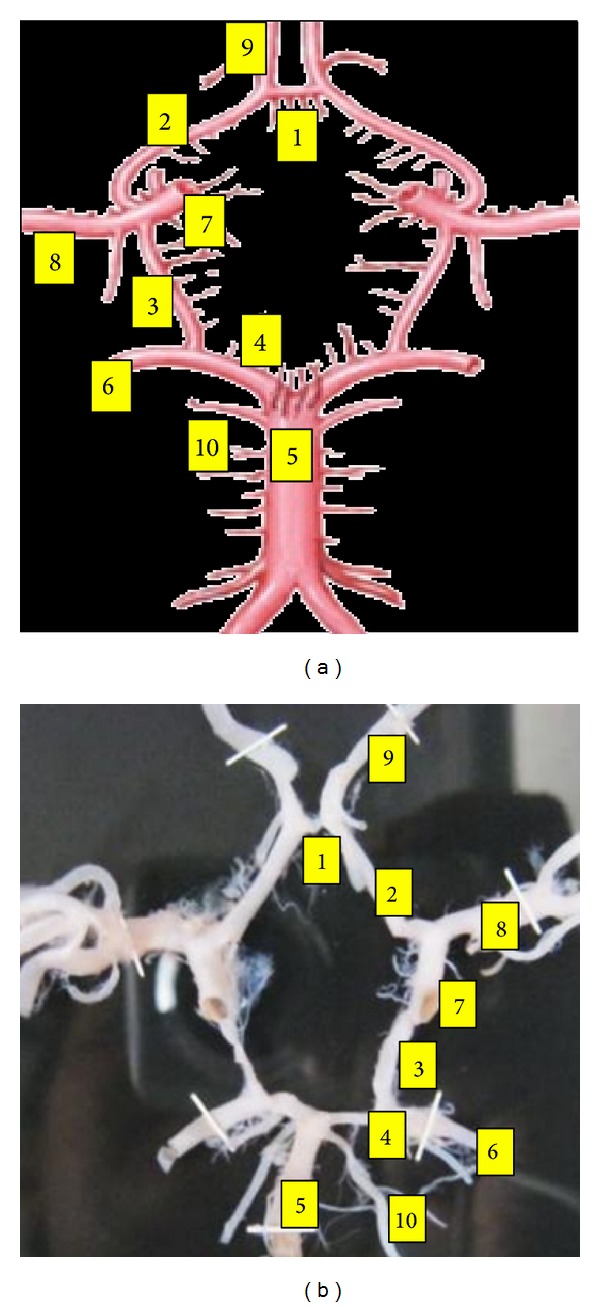
Classical circle of Willis. (1) ACoA, anterior communicating artery, (2) A1, precommunicating segment of anterior cerebral artery (ACA), (3) PoCA, posterior communicating artery, (4) P1, precommunicating segment of posterior cerebral artery (PCA), (5) BA, Basilar artery, (6) P2, postcommunicating segment of posterior cerebral artery (PCA), (7) ICA, internal carotid artery, (8) MCA, middle cerebral artery, (9) A2, postcommunicating segment of ACA, and (10) SCA, superior cerebellar artery.

**Table 1:** Morphological variations.

Variation type	Number of variations	Percentage %
Complete and normal CW	90	60
CW gross variations	60	40
Incomplete CW	16	10.66
ACoA	60	40
A1	21	14
PCoA	75	50
P1	21	14

**Table 2:** Morphometry of components of circulus arteriosus. (in millimeters).

	Right				Left			
	Min	Max	Mean	SD	Min	Max	Mean	SD
Length								
ACoA	1	20	2.6	0.21				
A1	8	25	17.8	0.34	10	25	17.4	0.33
PCoA	5	25	15.9	0.46	8	23	15.4	0.43
P1	5	20	7.5	0.30	5	15	7.6	0.28
Diameter								
ACoA	1	4	1.7	0.08				
A1	1	5	2.8	0.09	1	5	2.8	0.09
PCoA	1	4	2.1	0.12	1	4	2.1	0.12
P1	1	4	2.8	0.08	1	4	2.8	0.08
ICA	3	7	4.9	0.07	3	7	4.9	0.07
BA	3	6	4.9	0.06				

**Table 3:** Symmetry of different components of CW.

Segment of CW	Symmetrical	Asymmetrical
A1	103 (68.66%)	47 (31.33%)
PCoA	93 (62%)	57 (38%)
P1	116 (77.33%)	34 (22.66%)

**Table 4:** Percentages of different variations of CW.

Variations	Components	Number of variations	%	Total	Total percentage
Aplasia	ACoA	12	8	26	17.33
	A1	4	2.66		
	PCoA	6	4		
	P1	4	2.66		

Hypoplasia	ACoA	10	6.66	63	42
	A1	3	7.14		
	PCoA	41	27.33		
	P1	9	6		

Duplication	ACoA	16	10.66	28	18.66
	A1	9	6		
	PCoA	—	—		
	P1	3	2		

Fenestrations	ACoA	5	3.33	8	5.33
	A1	—	—		
	PCoA	1	0.66		
	P1	2	1.33		

**Table 5:** Comparison of morphological variations in percentage.

Variation type	Present study	Kapoor et al. [17]	Alpers et al. [12]	Fawcett and Blachford [18]	Hasebe and Adachi [19]
Complete classical CW	60	—	52.3	11	73
Incomplete CW	10.66	3.2	0.6	—	—
CW gross variations	40	54.8	—	—	—
ACoA	40	—	—	—	—
A1	14	—	—	—	—
PCoA	50	—	—	—	—
P1	14	—	—	—	—

**Table 6:** Comparison of morphological variations of ACoA in percentage.

Variation type	Present study	Kwak et al. [20]	Saidi et al. [21]	Baptista [22]	Kanemoto et al. [23]	Zhao et al. [24]	Uchino et al. [25]	de Almeida [26]	Perlmutter and Rhoton [27]	Gomes et al. [28]
Absent	8	—	—	—	—	—	—	—	—	—
Hypoplastic	10	—	—	—	—	—	—	—	—	—
Double	10.66	—	14	—	—	—	—	18	30	43.3
Fenestrations	3.33	—	2.6	—	—	0.8	1.2	—	—	—
3rd A2 seen	3.33	—	—	—	—	—	—	—	—	—
Aneurysm	8	5.7	—	13	13	—	—	—	—	—

**Table 7:** Comparison of morphological variations of A1.

Variation type	Present study	Al-Hussain et al. [29]	Riggs and Rupp [14]	Piganiol et al. [30]	Macchi et al. [31]
Absent	2.66	2	—	2.1	—
Hypoplastic	5.33	8	7	—	0.7
Duplication	6	10	—	—	—
Symmetrical	68.66	—	—	—	—
Asymmetrical	31.33	—	—	—	—

**Table 8:** Comparison of morphological variations of PoCoA.

Variation type PoCoA	Present study	Kapoor et al. [17]	Alpers et al. [12]	Al-Hussain et al. [29]	Merkkola et al. [32]
Absent	4	1	—	13	46%
Hypoplasia	27.33	—	—	—	—
Fenestration	0.66	—	—	33	—
PCA formation	18	—	14.6	77	—
Symmetrical	62	—	—	—	—
Asymmetrical	38	—	—	29	—

**Table 9:** Comparison of size of PoCoA.

Variation type	Adult type %	Foetal type %
Present study	77.33	18
de Vriese [7]	58	—
Riggs and Rupp [14]	76	17
Merkkola et al. [32]	50	—
Saeki and Rhoton [33]	70	30
Zeal and Rhoton[34]	58	40
Eftekhar et al. [35]	—	27

**Table 10:** Comparison of morphological variations of P1.

Variation type P1	Present study %	Kapoor et al. [17]	Alpers et al. [12]	Al-Hussain et al. [29]
Absent	2.66	—		—
Hypoplasia	6	10.6	6.3	1
Duplication	2	2.4		—
Fenestration	1.33	—		—
SCA + P1 stem	2	—		—
Symmetrical	77.33	—		—
Asymmetrical	22.66	—		28

**Table 11:** Comparison of morphometry of components of CW (in millimeters).

CW segments	Present study	Pai et al. [36]	Krabbe-Hartkamp et al. [37]	Moore et al. [38]
ACoA length	2.6	2.5	—	—
ACoA diameter	1.7	2.1	1.2	1.47
A1 length	17.8	14.6	—	—
A1 diameter	2.8	2.8	2.3	2.33
PCoA length	5.9	—	—	—
PCoA diameter	2.1	—	1.2	1.45
P1 length	7.5	—	—	—
P1 diameter	2.8	—	2	2.13
ICA diameter	4.9	—	3.6	4.72
BA diameter	4.9	—	3	3.17
